# Hybrid particle-wave Monte Carlo OCT simulation method provides a three orders of magnitude improvement in efficiency

**DOI:** 10.1364/BOE.572256

**Published:** 2025-12-04

**Authors:** Gijs Buist, Arjen Amelink, Johannes F. de Boer

**Affiliations:** 1 LaserLaB, Department of Physics and Astronomy, Vrije Universiteit, Amsterdam, The Netherlands; 2 Department of Optics, Netherlands Organisation for Applied Scientific Research, TNO, Delft, The Netherlands

## Abstract

The attenuation coefficient of biological tissue could serve as an indicator of structural and functional changes related to the onset or progression of disease. Optical coherence tomography (OCT) provides cross-sectional images of tissue up to a depth of a few millimeters, based on the local backscatter properties. Monte Carlo (MC) simulations are ideally suited to investigate and improve OCT attenuation coefficient extraction in confounding cases of (low-order) multiple scattering and inclusions such as blood vessels. However, current MC methods are time-consuming due to the OCT detection configuration rejecting many of the backscattered photons. In this work, we present two MC OCT detection models, a conventional photon detection model and a hybrid particle-wave detection model based on spherical waves from a photon’s last scatter position, and compare their simulation efficiency by comparing the SNR of the resulting depth profiles for equal conditions and number of simulated photons. We find a three orders of magnitude increase in the simulation efficiency for the hybrid particle-wave model compared to the conventional photon detection model. Both models show excellent agreement with single-scatter theory for weakly scattering samples. Additionally, we use the hybrid particle-wave model to simulate experimentally measured samples with 0.1 mm^−1^ ≤*µ*_*s*_ ≤ ~4.5 mm^−1^. The resulting simulated depth profiles show excellent agreement with the experimental depth profiles, even in those cases where the single-scatter theory model fails to describe the experimental depth profiles accurately.

## 1. Introduction

Optical coherence tomography (OCT) is an imaging technique that is widely used to visualise cross sectional tissue structure with high resolution in order to diagnose pathologies [1]. Standard OCT imaging measures the depth-resolved intensity backscattered from tissue providing qualitative intensity maps of tissue morphology, with no direct quantitative relationship to the underlying spatial variations in tissue optical properties. Quantitative imaging of the local tissue optical properties could provide useful additional information for medical diagnosis [2]; consequently, there is significant interest in the determination of quantitative information from OCT images, such as the local scatter or attenuation coefficient $\mu_{t}$ . Current state-of-the-art methods for depth-resolved $\mu_{t}$ quantification assume a Lambert-Beer decay of the OCT signal as a function of depth due to scattering and absorption, which is valid for singly scattered light [3,4]. Additionally, the OCT signal needs to be compensated for the depth dependent effect of the detection efficiency of a focused beam in the sample. This so-called confocal effect or confocal function has been derived assuming singly scattered light [5,6]. However, this current method for quantifying $\mu_{t}$ , that combines the confocal function with Lambert-Beer decay, is limited in its applicability to situations where single scattering dominates the OCT signal and, therefore, to weakly scattering samples (or superficial depths). In order to investigate the effect of (low order) multiple scattering on the determination of the confocal detection efficiency and the determination of the attenuation coefficient, Monte Carlo (MC) simulations are ideally suited. A number of groups have previously used Monte Carlo simulation to investigate OCT signals [7–10]. However, Monte Carlo simulations are time consuming, especially in an OCT configuration, where many simulated backscattered photons are rejected due to the confocal detection configuration. In this work we develop a framework for Monte Carlo OCT simulations that can investigate more robust and accurate $\mu_{t}$ extraction algorithms in those cases where the current single-scatter theory model fails. Two distinct detection models are developed, a conventional MC photon detection model and a hybrid particle-wave detection model based on spherical waves from a photon’s last scatter position. As expected, both MC simulation models are in good agreement with the single-scatter theory for weakly scattering samples but show a deviation from the single-scatter theory in stronger scattering samples due to multiple scattering contributions. By investigating the signal-to-noise ratio (SNR) as a function of the number of simulated photons we show that the hybrid particle-wave model is 3 orders of magnitude more efficient, providing a significant advantage over the conventional model. Finally, the MC simulations are validated by simulating four experimentally measured Intralipid dilution samples with scattering coefficients $\mu_{s}$ ranging from $\mu_{s}=0.1$ ${\mathrm{mm}}^{-1}$ to $\mu_{s}=4.5$ ${\mathrm{mm}}^{-1}$ . The simulated depth profiles show excellent agreement with the measured depth profiles for all four of the Intralipid dilutions while the single-scatter theory shows a large discrepancy with the measured depth profile for the Intralipid sample with the highest $\mu_{s}$ of 4.5 ${\mathrm{mm}}^{-1}$ .

## 2. Monte Carlo simulation of OCT

We developed OCT depth profile simulations using the Monte Carlo eXtreme (MCX) software package [11] to simulate light transport, or radiative transfer, for user defined samples using discrete energy packages called photons. To generate OCT depth profiles from MCX photon output we introduce two detection models, one using the particle-like photon properties which we call the conventional model and one where we replace backscattered photons with spherical waves originating from their last scatter position in the sample which we call the hybrid particle-wave model.

### 2.1. Monte Carlo simulations

**Fig. 1.**
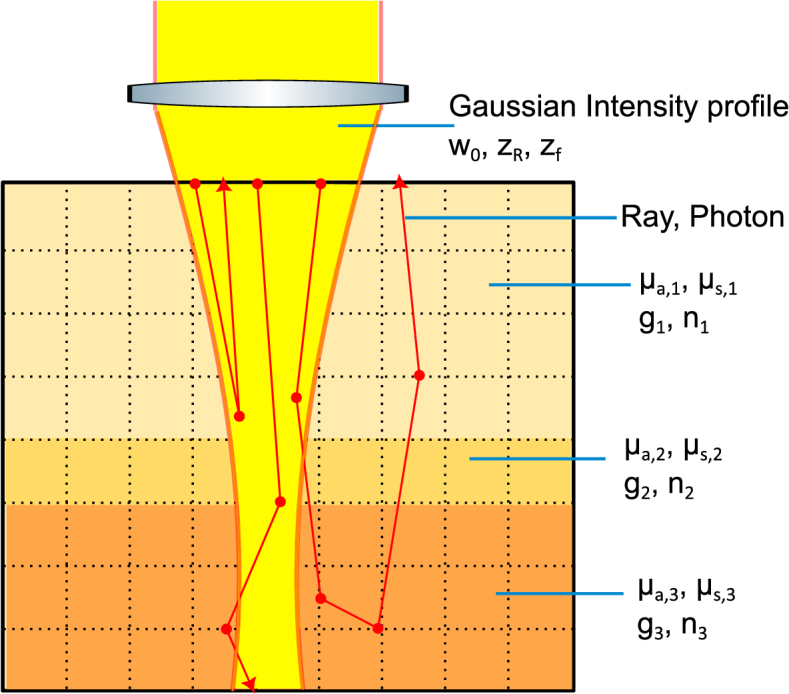
Cross-section example of MCX simulation. Rectangle with black bounding box indicating cross-section of simulated volume with 3 distinct colours showing different media types and therefore different optical properties. Black dotted lines forming squares represent voxels within which optical properties are defined in the simulation. The yellow beam indicates the Gaussian intensity distribution in case of no interaction (scattering or absorption) determining how the photons will be launched. The three paths shown by the red lines represent examples of possible photon paths with, from left to right, a singly (back)scattered photon, a transmitted photon and a backscattered photon that underwent multiple scattering.

To simulate light transport, or radiative transfer, in scattering media the Monte Carlo eXtreme (MCX) software package [11] propagates light in discrete photon packets, which move along straight lines and interact with a user defined simulated volume based on four optical parameters defined for each voxel in the volume. These parameters are the absorption coefficient ( $\mu_{a}$ ), which determines the absorption along a photon packet path, the scattering coefficient ( $\mu_{s}$ ), which determines the scatter probability (or distance travelled between scatter events), the phase function, which determines the scatter angle, and finally, the refractive index n . In this work, the Henyey-Greenstein phase function, which depends on the scattering anisotropy ( g=<cos⁡θ> ), was used. Absorption is not modelled as a binary probability, but instead reduces the weight of a photon package along the path travelled through the medium. To simulate a focused Gaussian beam with a beam waist $w_{0}$ (defined as the $1/e^{2}$ radius of the lateral intensity distribution), a Rayleigh range of $z_{R}$ and focus position $z_{f}$ , we used the method described by Tycho *et al*.[9,12]. They showed that for a Gaussian beam, surfaces of constant intensity are hyperboloids of one sheet which can be fully defined with straight lines as it is a doubly ruled surface. As a result, photons can be launched in straight lines while still generating a Gaussian intensity profile. A sketch of an example MCX simulation cross-section is presented in Fig. 1.

### 2.2. OCT detection

OCT is a low-coherence interference detection method where an interference signal between light backscattered from a sample and light from a reference arm is measured as a function of the optical pathlength (OPL) difference between the light from the two arms. Assuming the backscattered light to have travelled only along the optical axis, so negligible lateral distance travelled, the OPL of the backscattered signal can be related to the depth if the refractive index along the depth is known. This allows for the OCT signal to be interpreted as a depth-resolved signal and the visualisation of tissue morphology or sample structure.

To simulate OCT signals the round trip OPLs of the backscattered photons through the simulated volume are saved. The photons are assigned to OPL bins according to a coherence gate, and their detected intensities are determined via either the conventional or hybrid particle-wave method. These intensities are then incoherently summed per OPL bin to get the OPL resolved OCT signal. So the OCT intensity at OPL bin j is given by [13,14]

(1) $$I_{j}=\sum_{i}I_{\text{det},i,j},$$

with $I_{\text{det},i,j}$ being the detected intensity to be determined by the conventional or hybrid particle-wave models, discussed in the next sections, for the i ’th photon in the j ’th OPL bin.

**Fig. 2.**
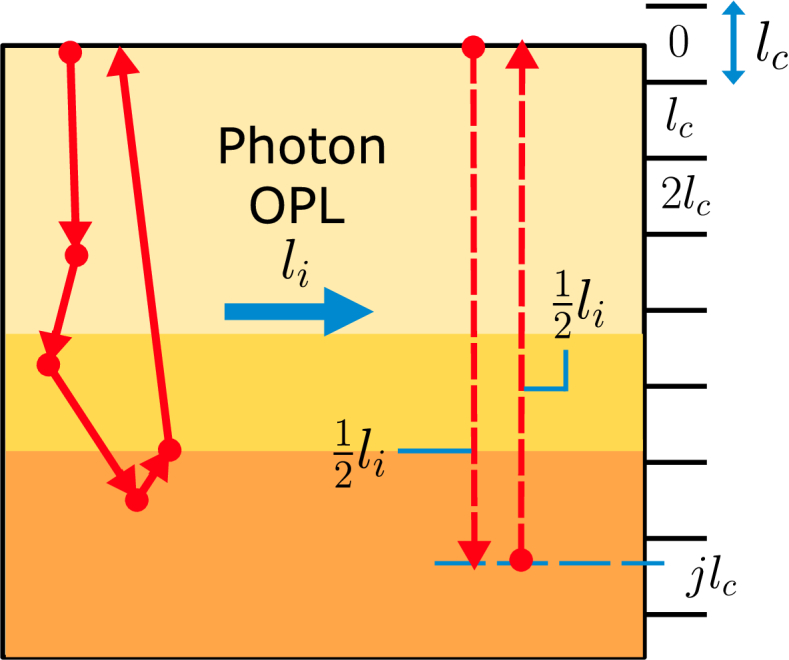
Sketch of the optical pathlength (OPL) binning of a backscattered photon. From the MCX simulation a round trip OPL $l_{i}$ of the simulated photon is extracted based on its (physical) simulated path (solid red line with arrows) and refractive index of the simulated medium along its path. The red dashed lines represent the one-way photon OPL, or $\frac{1}{2}l_{i}$ , which is used together with the OPL depth bin resolution $l_{c}$ to assign the photon to depth OPL bin $jl_{c}$ . The OPL depth $jl_{c}$ can be converted to a physical depth if the refractive index along the depth is known. The $l_{c}$ and max OPL depth depend on the OCT system used/simulated. Note that lateral travel increases perceived OPL depth and therefore the associated physical depth.

A schematic representation of OPL binning of a photon is shown in Fig. 2. A photon with round trip OPL $l_{i}$ is assigned to an OPL bin j if the following inequality is satisfied

(2) $$\left|\frac{1}{2}l_{i}-jl_{c}\right|<\frac{1}{2}l_{c},$$

with $l_{c}$ being the one-way trip OPL bin resolution and the factor one-half in front of $l_{i}$ to convert it from round trip to one-way trip. The one-way trip $l_{c}$ , or depth resolution of an OCT system, is given by the coherence length of the source. Throughout this work a $l_{c}=4$ μ m was used for the simulations. Finally, to convert OPL to depth we know that for an arbitrary physical path s the associated OPL is

(3) OPL(s)=∫n(s)ds,

with n(s) being the refractive index along s . With, in the case of OCT, the path s assumed to be a round trip path fully along depth z . If the refractive index is constant along the probed depth z , as will be the case throughout this work, the depth $z_{j}$ associated with *one-way trip* OPL bin j is given by

(4) $$z_{j}=\frac{jl_{c}}{n},$$

with n being the (constant) refractive index.

**Fig. 3.**
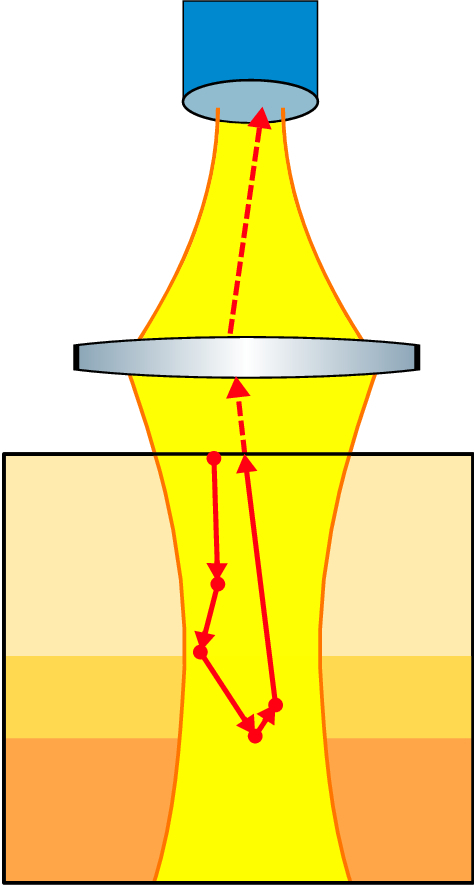
Sketch of the conventional detection model for a single photon. MCX simulates a volume and the path a photon travels through it, photons that scatter back trough the simulated volume surface are saved, solid red arrows. The backscattered photons are then propagated, red dashed line, to a SMF detector under the assumption that the SMF functions as both the source and detector as indicated by the yellow beams.

#### 2.2.1. Conventional model detection scheme

In the conventional MC detection model the position and direction of backscattered photons exiting the simulated volume are used to propagate the photons to the surface of the single mode fibre (SMF) detector (see Fig. 3 and Appendix A.1). The detected photon intensity (weight) is calculated based on the photons intensity (weight) $I_{i}$ from the MCX simulation and its position and direction at the SMF surface ( $I_{\text{det, conv},i}=W_{\text{pos}}W_{\text{dir}}I_{i}$ ). We determine $W_{\text{pos}}$ based on the lateral position amplitude of the SMF mode and $W_{\text{dir}}$ based on the angular direction amplitude of an SMF mode. The SMF mode is approximated as a Gaussian beam at the SMF surface. Note that both $W_{\text{pos}}$ and $W_{\text{dir}}$ are field (amplitude) coupling coefficients so that their product, $W_{\text{pos}}W_{\text{dir}}$ , is an intensity coupling coefficient that incorporates both a lateral position and an angular dependence.

Given a Gaussian SMF mode with beam waist radius $w_{0}$ , Rayleigh range $z_{R}$ and with its focus at the SMF surface, the amplitude position weight of a photon on the SMF surface is then given by

(5) $$W_{\text{pos}}=\exp\left(-\frac{x^{2}+y^{2}}{w_{0}^{2}}\right),$$

with x and y being coordinates in the lateral plane whose origin sits at the centre of the SMF.

The amplitude angle weight of the photon impinging on the SMF surface, which is equivalent to the amplitude coupling efficiency of a plane wave with a Gaussian SMF mode, is given by

(6) $$W_{\text{dir}}=\exp\left(-\frac{w_{0}^{2}}{4}(k_{x}^{2}+k_{y}^{2})\right),$$

with $k_{x}$ , $k_{y}$ being the x and y component of the wave vector k . Using definitions $z_{R}=\frac{1}{2}kw_{0}^{2}$ and $\left(k_{x},k_{y},k_{z})=k(v_{x},v_{y},v_{z}\right)$ , with $v_{i}$ being components of the normalised directional vector, it can be rewritten in terms of the Gaussian beam parameters and the directional vector of the photon as

(7) $$W_{\text{dir}}=\exp\left(-\frac{z_{R}^{2}}{w_{0}^{2}}(v_{x}^{2}+v_{y}^{2})\right),$$

which can be further simplified using the divergence angle of a Gaussian beam, $\theta_{\text{div}}=w_{0}/z_{R}$ to

(8) $$W_{\text{dir}}=\exp\left(-\frac{v_{x}^{2}+v_{y}^{2}}{\theta_{\text{div}}^{2}}\right).$$

So finally, in the conventional model we obtain the intensity detected by the SMF from photon i with intensity weight $I_{i}$ , position $\left(x_{i},y_{i}\right)$ on the SMF surface and lateral directional vector $\left(v_{x,i},v_{y,i}\right)$ at the SMF surface as the product of the amplitude probability for position and lateral directional vector, given by

(9) $$I_{\text{det, conv},i}=I_{i}\exp\left(-\frac{x_{i}^{2}+y_{i}^{2}}{w_{0}^{2}}\right)\exp\left(-\frac{v_{x,i}^{2}+v_{y,i}^{2}}{\theta_{\text{div}}^{2}}\right).$$

#### 2.2.2. Hybrid particle-wave method detection scheme

**Fig. 4.**
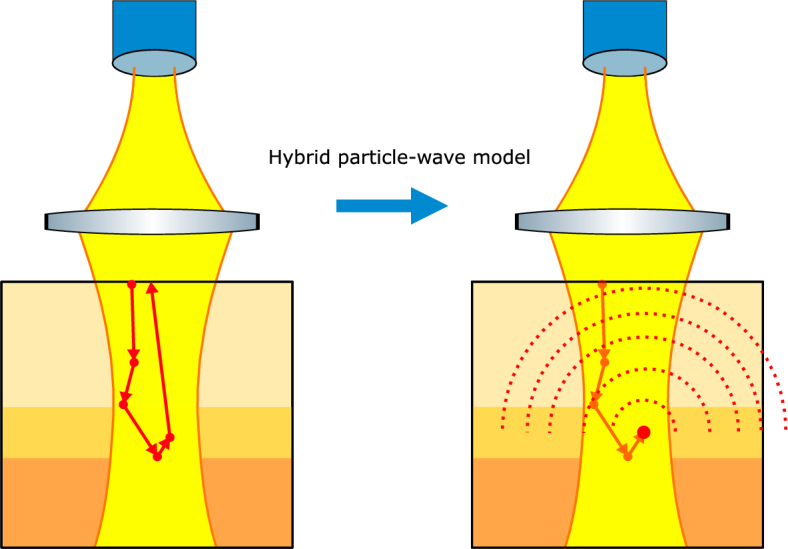
The hybrid particle-wave model. On the left the standard situation of the MCX simulations with the simulated volume, the backscattered photon path in solid red arrows and the yellow beams indicating the Gaussian SMF mode. On the right a representation of the hybrid particle-wave model. The last stretch of the photon path from the last scatter position to the surface has been replaced with a spherical wave as indicated by the red dashed spherical wavefronts. The path of the photon from its launch into the simulated volume up to the last scatter site has been made more transparent for clarity.

For the hybrid particle-wave model we use the same standard MCX simulation as for the conventional model, but at the location of the last scatter position before exiting the medium the photon is replaced by a spherical wave, see Fig. 4.

We need to consider carefully how the detected intensities and OPL are affected when moving from the pure photon particle picture to the hybrid particle-waves picture. The photon leaving the sample can be envisioned as a sampling of the spherical wave probability, or one realization in the MC simulation of all the possible photon paths generated by the spherical wave. To this end we need to take into account the following four phenomena, A) the power coupling coefficient T of the spherical wave with the SMF, calculated by the absolute value squared of the overlap between the spherical wave E-field and SMF (field) mode. B) Energy conservation needs to be satisfied. Consider the intensity flux of a spherical wave through a half sphere and through a flat surface. The flux should be the same, which requires projecting the intensity flux onto the surface normal. In analogy, the energy flux of a simulated photon through the sample surface is given by the energy of the photon multiplied with the cosine of the angle between the photon incident on the surface and the surface normal. C) Envisioning the measured photon as a sampling of the spherical wave probability, one needs to realize that the sampling of this spherical wave needs to be corrected for the attenuation of this photon along the photon path. Simulated photons that travelled at an angle towards the sample surface have traversed a longer path from the last scatter location to the surface and have encountered a larger probability to be attenuated along that path. The attenuation along this longer path results in an underestimation of the sampling of the spherical wave probability. To correct for this effect, the probability is multiplied with an exponential correction factor proportionally to the longer optical path to the surface. D) The optical path needs to be estimated properly for correct OPL binning. When replacing photons with spherical waves from their last scatter positions the final pathlength from photons with identical last scatter positions should be identical since they are replaced by the same spherical wave. Taking into account the optics that focuses the exiting spherical wave from the tissue to the detector, all optical paths from a spherical wave to the image are equal in length, which we represented with the shortest path to the surface from the last scatter position.

**Fig. 5.**
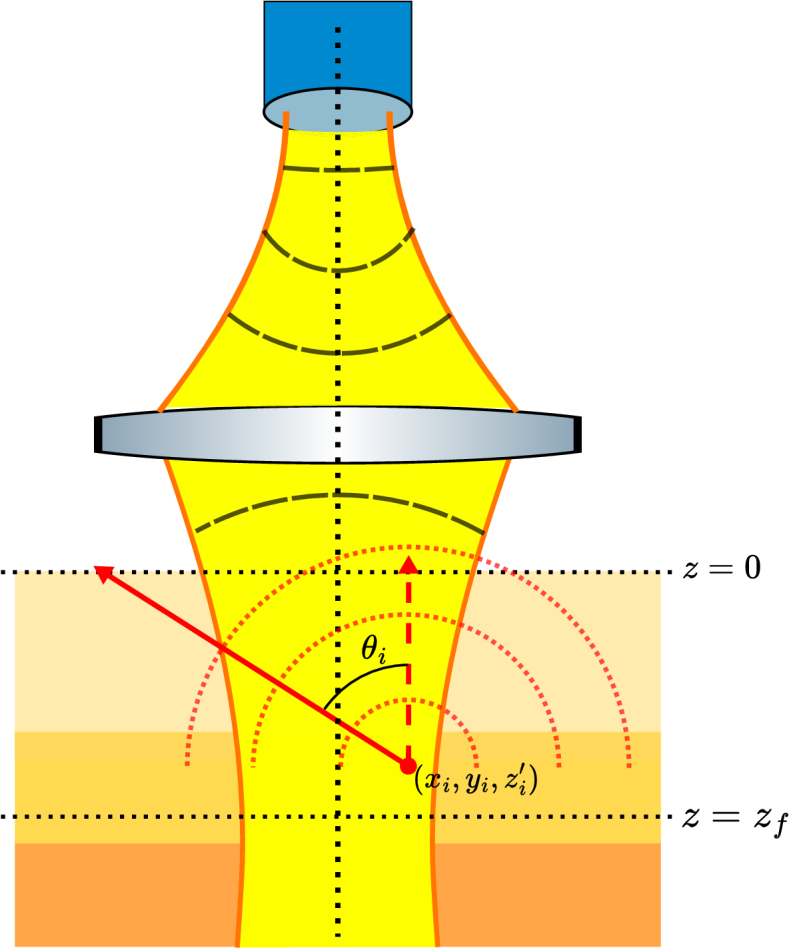
Sketch of the hybrid particle-wave model showing how to connect the backscattered photon particle view to that of the spherical wave. The last scatter position of the backscattered photon is indicated by a red dot, $\left(x_{i},y_{i},z_{i}^{\prime }=z_{i}-z_{f}\right)$ , which, together with the SMF Gaussian mode, indicated with the yellow beam, is used to calculate power transmission coefficient at the plane z=0 (term A) in the main text). The red dotted curves and black dashed curves show the phase fronts of the spherical wave and the SMF Gaussian mode, respectively. The solid red arrow indicates the MCX simulated photon path from the last scatter event to the sample surface while the red dashed line shows the shortest path from the last scatter position to the sample surface, i.e. depth of last scatter event. The angle between the solid and dashed arrows is the incident angle of the backscattered photon with respect to the surface normal and is used to calculate its flux trough the sample surface (term B) in the main text). The attenuation along the solid and dashed red paths are used to calculate the intensity of the spherical wave based on the intensity of the backscattered photon ( term C) in the main text). The OPL along solid and dashed red paths are used to correct the total round trip OPL used for OPL binning ( term D) in the main text). The path of the photon from its launch in the simulation to its last scatter position is not shown for clarity.

Figure 5 schematically shows the setup for the hybrid model detection in a cross-section, with z being the distance from medium surface to last scatter position and $z^{\prime }=z-z_{f}$ is the axial distance between the last scatter position and the focus of the Gaussian beam in the simulated volume. These aforementioned four effects are calculated below.

A) The power transmission coefficient T of a single spherical wave with the (Gaussian) mode of the SMF detection fibre is given by,

(10) $$T=\eta_{c}\eta_{c}^{*}=\frac{{\left|\iint E_{SMF}^{*}(r_{⊥})E(r_{⊥})d^{2}r_{⊥}\right|}^{2}}{\iint {\left|E_{SMF}(r_{⊥})\right|}^{2}d^{2}r_{⊥}\iint {\left|E(r_{⊥})\right|}^{2}d^{2}r_{⊥}},$$

where $\eta_{c}$ is the coupling efficiency and $\eta_{c}^{*}$ is its complex conjugate. $E_{SMF}$ is the SMF mode which we approximate with a Gaussian beam and E is the field of the spherical wave. We refer to Appendix A.2 for the calculation of T . Using a Gaussian beam for the SMF mode with focus at $\left(0,0,z_{f}\right)$ and a spherical wave at (x,y,z) this results in a power transmission coefficient,

(11) $$T(x,y,z^{\prime })={\left(\frac{w_{0}}{z_{R}}\right)}^{2}\frac{w_{0}^{2}}{w{\left(z^{\prime }\right)}^{2}}e^{-2\frac{x^{2}+y^{2}}{w{\left(z^{\prime }\right)}^{2}}}\text{, with }w(z^{\prime })=w_{0}\sqrt{1+{\left(\frac{z^{\prime }}{z_{R}}\right)}^{2}},$$

and $z^{\prime }$ is the axial distance to the focus, $z^{\prime }=z-z_{f}$ .

B) The flux of the backscattered photon through the sample surface is calculated according to

(12) $$I_{i}\cos(\theta_{i}),$$

where $I_{i}$ is the backscattered photon intensity weight and $\theta_{i}$ its incident angle with respect to the surface normal.

C) The correction factor accounting for underestimating the intensity weight of the spherical wave due to attenuation along the longer paths to the surface is calculated as follows. The attenuation along the actual path s is given by $\exp\left[-\int \mu_{t}(s)ds\right]$ , the attenuation along the shortest path for the spherical wave is given by $\exp\left[-\int \mu_{t}(z)dz\right]$ , with z being the path of the spherical wave which is the shortest distance from the last scatter position to the surface of the simulated volume. By taking the ratio of the attenuation along the shortest path over the attenuation along the photon path we get the adjustment factor shown below:

(13) $$P_{\text{adj}}=\frac{\exp\left[-\int \mu_{t}(z)dz\right]}{\exp\left[-\int \mu_{t}(s)ds\right]}.$$

The attenuation coefficient $\mu_{t}=\mu_{a}+\mu_{s}$ along the paths include both absorption and scattering. In case of constant $\mu_{t}$ this can be simplified to,

(14) $$P_{\text{adj}}=\frac{e^{-\mu_{t}z}}{e^{-\mu_{t}z/\cos(\theta )}}=e^{\mu_{t}z\left(1/\cos(\theta )-1\right)},$$

where z is distance to the surface and θ is the incident angle on the sample surface of the backscattered photon with respect to the surface normal.

Combining terms A) through C) we get the detected intensity from a single photon in the hybrid model

(15) $$I_{\text{det, hyb},i}=I_{i}\cos(\theta_{i})T(x_{i},y_{i},z_{i}^{\prime })e^{\mu_{t}z_{i}\left(1/\cos(\theta_{i})-1\right)},$$

with on the right hand side of the equation from left to right, the intensity of the backscattered photon $I_{i}$ , the photon flux perpendicular to the surface $\cos(\theta_{i})$ , the power transfer function of a spherical wave from the last scatter position T and the probability adjustment term for going from photon to spherical wave.

Finally D), we need to reconsider the (optical) pathlength (bins) associated with the detected intensity of the photons with a spherical wave at the last scatter position. Spherical waves from the same last scatter position should give an identical pathlength segment regardless of the photon path from the last scatter position. So the pathlength of the last trajectory needs to be replaced with the pathlength of the spherical wave, which is the shortest distance to the surface. A new round trip OPL is calculated by taking the original full round trip OPL and subtracting the difference between the photon OPL from the last scatter position to the simulated volume surface and the spherical wave OPL from the last scatter event to the surface, with the OPL of the spherical wave calculated along the shortest path z from the last scatter position to the surface of the simulated volume, see Fig. 5. The new round trip OPL $l_{i}^{\prime }$ is

(16) $$l_{i}^{\prime }=l_{i}-l_{\Delta ,i},$$

with $l_{i}$ being the original round trip OPL and $l_{\Delta ,i}$ the change in OPL due to a spherical wave at the last scatter position. In case of constant refractive index $l_{i}^{\prime }$ becomes,

(17) $$l_{i}^{\prime }=l_{i}+nz_{i}\left(1-\frac{1}{\cos(\theta_{i})}\right),$$

where this $l_{i}^{\prime }$ will be used in Eq. (2) instead of $l_{i}$ to determine the OPL bin.

## 3. Methods

### 3.1. OCT measurements

To validate the simulation methods a selection of previously published [5] OCT measurements of different Intralipid dilution samples were simulated. The OCT measurements were performed with a SMF based swept source (SL132120, Thorlabs) OCT (SS OCT) device with a central wavelength of 1300 nm, bandwidth of 90 nm, a sweep rate of 200 kHz and a depth scan range and depth resolution $l_{c}$ of 8 mm in air and 11 μm in tissue, respectively. In the sample arm a beam was outcoupled from the SMF (SMF-28-J9, Thorlabs), collimated with a reflective collimator (RC04APC-P01, Thorlabs), directed by a Galvo mirror scanning system (GVS002, Thorlabs) and finally focused (SL50-3P - Scan Lens, Thorlabs) resulting in a Gaussian beam with $w_{0}=15$ μm and $z_{R}=750$ μm in the sample or $z_{R,\text{air}}=564$ μm in air. To reduce noise an average depth profile from 250000 individual depth profiles (A-lines) was calculated and processed as described in [5]. The focus position $z_{f}$ of the Gaussian beam for each measurement was extracted based on the confocal function fits on the focus series measurement as explained in [5]. The nominal $\mu_{s}$ and g at λ=1300 nm for the different Intralipid solutions according to Aernouts *et al*. [15,16] in case of dependent and independent scattering are given in Table 1 below. Independent scattering is scattering due to a particle that is unaffected by the presence of any other particle and therefore the scattering coefficient scales linearly with scatterer concentration. In case of dependent scattering, the scattering of a single particle is affected by (nearby) other particles which leads to a non-linear relationship between $\mu_{s}$ and scatterer concentration. In the wavelength range of the OCT system the absorption of the oil droplets in Intralipid is negligible [17] which is why the absorption coefficient of water was used, $\mu_{a}=0.15$ ${\mathrm{mm}}^{-1}$ [18]. The refractive index of the Intralipid dilutions was assumed to be equal to that of water, n=1.33 [16,17] and along the sample beam path there is an air-sample interface leading to a critical angle for total reflection of

(18) $$\theta_{C}=\arcsin\left(\frac{n_{\text{Intra}}}{n_{\text{air}}}\right)=49^{\circ }.$$

Which in the simulation was approximated by a maximum incident angle on the sample interface of $\theta_{i}<50^{\circ }$ .

**Table 1.** Properties Intralipid dilutions compared to stock Intralipid 20% (IL20). Both Independent and dependent scattering coefficients $\mu_{s}$ and scatter anisotropies g based on Aernouts *et al*. [15,16]

Label	I01	I1	I4	I10	Stock IL20

stock IL 20 solution volume fraction	0.006	0.062	0.249	0.622	1
Scatterer conc. [vol%]	0.1	1.4	5.7	14.1	22.7
nominal $\mu_{s}$ [ ${\mathrm{mm}}^{-1}$ ] independent scat	0.1	1	4	10	16
nominal $\mu_{s}$ [ ${\mathrm{mm}}^{-1}$ ] dependent scat	0.1	1	3.2	5.5	6
nominal g independent scat	0.33	0.33	0.33	0.33	0.33
nominal g dependent scat	0.33	0.33	0.30	0.24	0.18

For samples I4 and I10 the optical properties given by Aernouts *et al*. [16] resulted in simulated depth profiles that appeared to be imperfect fits for the experimentally measured depth profiles. Therefore a series of simulations with different $\mu_{s}$ and g around the Aernouts dependent scattering values were performed and the agreement between the resulting simulated depth profiles and experimentally measured depth profiles was assessed. To quantify the fit quality first the simulated depth profiles were fitted to the experimentally measured profiles as described in Section 3.2, after which the root-mean-square deviation (RMSD) between the (fitted) simulated depth profile and experimentally measured depth profile was calculated as,

(19) $$\text{RMSD}(I_{\text{sim}})=\sqrt{\frac{\sum_{j=1}^{M}{\left(I_{\text{sim}}(z_{j})-I_{\text{exp}}(z_{j})\right)}^{2}}{M}},$$

with j the depth pixel index (see Eq. (4)) and M total number of pixels. The $\mu_{s}$ and g combination that resulted in the fitted simulated depth profile with the lowest RMSD was considered as the actual or best approximation of the optical parameters of the Intralipid dilution.

### 3.2. Fitting procedures

The simulated depth profiles of both detection models were compared to a simple model describing the OCT measured backscatter intensity called the single-scatter model. This model assumes that only singly scattered light contributes to the OCT signal and that the same SMF functions as both the source and the detector of the illumination and backscattered beams, respectively. From these assumptions the following expression is derived for a homogeneous medium [5]

(20) $$I_{\text{theory}}(z)=\frac{A}{1+{\left(\frac{z-z_{f}}{z_{R}}\right)}^{2}}e^{-2\mu_{t}z},$$

where $\mu_{t}$ is the attenuation coefficient which is the sum of the absorption $\mu_{a}$ and scattering $\mu_{s}$ coefficients ( $\mu_{t}=\mu_{a}+\mu_{s}$ ), $z_{f}$ is the physical focus position of the illumination beam in the sample, $z_{R}$ is the Rayleigh range in the sample of the illumination beam, z is the physical depth from the sample surface and A is a normalisation constant. In this equation, the fractional first factor is related to the SMF confocal detection geometry (and called the confocal function) while the exponential second factor is related to the Lambert-Beer decay as a function of depth.

For the simulations the optical properties and beam parameters are known within numerical precision and therefore in the theory-simulation comparison there can only be a difference of a constant factor A when the theory is valid, i.e. weak scattering. The fit was performed by minimizing an L2 norm,

(21) $$\text{ min}\langle {\left[I_{\text{sim}}(z)-AI_{\text{theory}}(z)\right]}^{2}\rangle .$$

The above equation was evaluated using least squares with the curve_fit method from the Python package of Scipy (scipy.optimize.curve_fit).

To compare the simulated depth profiles with the experimentally measured depth profiles the experiment intensity values first needed to be interpolated to the sample depths of the simulated depth profiles. Additionally, the experimentally measured depth profiles have a noise floor due to shot-noise and thermal noise which is an effect not present in the simulations and especially affects strongly attenuation samples. To take this into account an additional term C is added to the fit. In order to accurately fit the depth profiles including the noise floor, which can be hampered by the decreased sensitivity to (relatively) low signal values in a least square fit method, we used Eq. (22):

(22) $$\text{min}\left\langle {\left[\frac{AI_{\text{sim}}(z)+C}{I_{\text{exp}}(z)}-1\right]}^{2}\right\rangle .$$

When fitting the theory to the experiment the same fit as above is performed with $I_{\text{sim}}$ replaced with the single-scatter theory $I_{\text{theory}}$ of Eq. (20), with $z_{f},z_{R}$ and $\mu_{t}$ fixed to the values used in the simulations.

### 3.3. NRMSD performance metric

To measure and compare the performance of the hybrid and conventional detection models a metric for the signal-to-noise ratio (SNR) of the simulated depth profiles as a function of the number of simulated photons is needed. The single-scatter theory curve was not used as the ground truth for the depth profiles since it is unreliable for more strongly scattering samples. Instead we generated depth profiles with negligible statistical noise for both detection models by running simulations with an extremely large amount of photons: in the order of $10^{11}$ to $10^{12}$ , which we considered to be the ground truth. Then multiple series of simulations with much smaller, but increasing, number of simulated photons for both methods were performed and subsequently compared to the ground truth depth profiles to quantify the noise on the depth profiles as a function of the number of simulated photons.

Since the intensity signal is proportional to the number of simulated photons, the ground truth depth profiles need to be rescaled or normalised based on the ratio of the number of simulated photons N and the number of photons in the ground truth $N_{\text{gt}}$ :

(23) $$A_{N}=\frac{N}{N_{\text{gt}}}.$$

$A_{N}$ is the normalisation constant for the ground truth depth profile to a depth profile with N simulated photons. Thereby the ground truth intensity depth profile is always scaled to the intensity of the simulated depth profile with statistical noise. Finally, noise was quantified using the normalized root mean squared deviation (NRMSD) between the simulated depth profile and the rescaled ground truth, with normalisation by the total intensity $\left(\sum_{j=1}^{M}A_{N}I_{\text{gt}}(z_{j}\right))/M$ , i.e.,

(24) $$\text{NRMSD}(N)=\frac{M}{\sum_{j=1}^{M}A_{N}I_{\text{gt}}(z_{j})}\sqrt{\frac{\sum_{j=1}^{M}{\left(I_{\text{sim}}(N,z_{j})-A_{N}I_{\text{gt}}(z_{j})\right)}^{2}}{M}}.$$

This NRMSD should then be proportional to the inverse of the SNR which in turn is expected to be proportional to the square root of the number of simulated photons, $\text{SNR}\propto \sqrt{N}$ . Therefore the conventional and hybrid detection model NRMSD( N ) signals can be fitted to,

(25) $$\text{NRMSD}(N)=\frac{\alpha }{\sqrt{N}},$$

where α is a proportionality constant. By fitting α in Eq. (25) to the NRMSD curves the simulation efficiency between the detection models can be quantified by the ratio of simulated photons that give equal NRMSD for both of the detection models. Specifically, the square of the ratio of the proportionality factors $\alpha_{C}$ and $\alpha_{H}$ for the conventional and hybrid models respectively, ${\left(\alpha_{C}/\alpha_{H}\right)}^{2}$ , is equal to the ratio of the number of simulated photons between the conventional and hybrid model respectively, ( $N_{C}/N_{H}$ ), needed for equal NRMSD and therefore equal SNR.

## 4. Results

### 4.1. Simulation models compared to theory

**Fig. 6.**
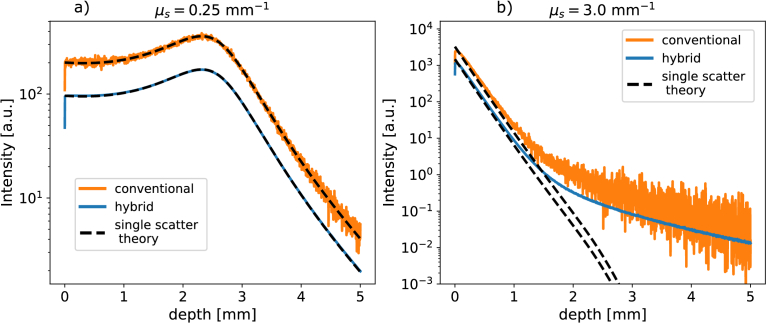
Simulated depth profiles for a weakly scattering medium, **a**, and a more strongly scattering medium, **b**, using the conventional detection model (orange lines) and the hybrid particle-wave detection model (blue lines). The black dashed lines indicate single-scatter theory curves which were fitted with only the normalisation constant as a free parameter. For both media the simulation parameters used were $w_{0}=15$ μ m, $z_{R}=750$ μ m and $z_{f}=2.57$ mm as beam parameters and $\mu_{a}=0.15$ ${\mathrm{mm}}^{-1}$ , g=0.3 and n=1.33 as optical properties. For weakly scattering medium **a**, $\mu_{s}=0.25$ ${\mathrm{mm}}^{-1}$ was used and $1.2\times 10^{11}$ photons were simulated for both detection models. For the more strongly scattering medium **b**, $\mu_{s}=3.0$ ${\mathrm{mm}}^{-1}$ and $2.4\times 10^{9}$ simulated photons were used for both detection models.

Figure 6(a)-(b) show, for a weakly and strongly scattering medium respectively, the simulated depth profiles for the conventional model in solid orange lines and for the hybrid particle-wave model in solid blue lines, as well as the single-scatter theory fitted to each depth profile in dashed black lines. In Fig. 6 the results for two different sample optical properties are shown with the simulated optical properties and beam parameters given in the caption. This choice of parameters is related to the experimental setup and samples used for the experimental validation of our models in Section 4.3.

The weak scattering case, Fig. 6(a), clearly shows that both conventional and hybrid detection models reproduce the expected single-scatter theory curve, with a stronger but also noisier signal for the conventional detection model compared to the hybrid particle-wave detection model. The more strongly scattering case, Fig. 6(b), shows that at lower depths the single-scatter theory curve fits well, but as the depth increases the simulated depth profiles deviate from single-scatter theory. This behaviour is expected as the amount of multiple scattered signal, which is unaccounted for by the single-scatter theory model, increases with depth (and with $\mu_{s}$ ). Additionally, similar to the weak scattering case, we see that the signal for the conventional model is stronger but at the same time much noisier compared to the hybrid particle-wave model.

### 4.2. Simulation models performance comparison

**Fig. 7.**
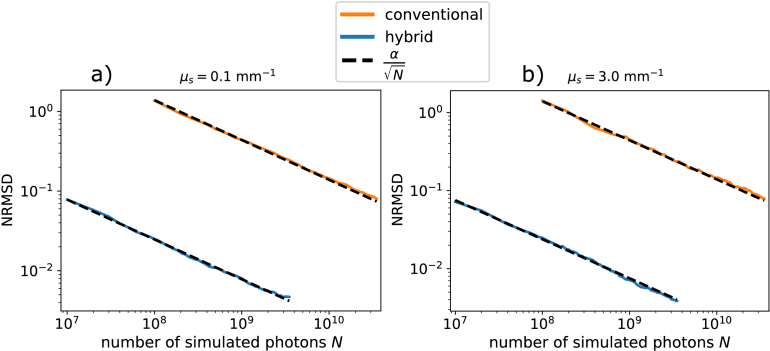
The NRMSD of simulated depth profiles as function of number of simulated photons for a weakly scattering medium, **a**, and a more strongly scattering medium, **b**. The solid orange line in the top of both figures indicates the NRMSD for the conventional model while the solid blue line in the bottom of both figures indicate the NRMSD for the hybrid particle-wave model, the black dashed lines indicate $\frac{\alpha }{\sqrt{N}}$ fits for each curve. For both media the simulated beam parameters were $w_{0}=15$ μ m, $z_{R}=750$ μ m and $z_{f}=2$ mm and simulated optical properties were g=0.3 and n=1.33 . For the weakly scattering medium **a**, $\mu_{a}=0.1$ ${\mathrm{mm}}^{-1}$ and $\mu_{s}=0.1$ ${\mathrm{mm}}^{-1}$ were used. The fitted α values were 13924 and 248 for the conventional and hybrid curves, respectively. For the more strongly scattering medium **b**, $\mu_{a}=0.15$ ${\mathrm{mm}}^{-1}$ and $\mu_{s}=3.0$ ${\mathrm{mm}}^{-1}$ were used. The fitted α values were 14010 and 237 for the conventional and hybrid curves, respectively.

To quantify the noise of the depth profiles as a function of simulated photons the normalized root mean square deviation (NRMSD) between simulated and ground truth depth profiles was calculated as described in Section 3.3. This NRMSD is shown to be inversely proportional to the SNR. Figure 7 shows the NRMSD as a function of the number of simulated photons N for a weakly scattering medium (Fig. 7(a)) and for a more strongly scattering medium (Fig. 7(b)). In both figures the solid orange line indicates the NRMSD for the conventional model while the solid blue line indicates the NRMSD for the hybrid particle-wave model; the black dashed lines indicate $\frac{\alpha }{\sqrt{N}}$ fits for each curve.

Figure 7 shows that the NRMSD of the conventional model is significantly higher than that of the hybrid model in both weakly and strongly scattering cases, which means that the conventional model SNR is significantly lower compared to the hybrid model for the same number of simulated photons.

Based on the $\frac{\alpha }{\sqrt{N}}$ fits to the NRMSD curves we calculate ${\left(\alpha_{C}/\alpha_{H}\right)}^{2}$ , with $\alpha_{C}$ and $\alpha_{H}$ the fitted values for the conventional and hybrid models, respectively, to find the simulated photon ratio that yield equal NRMSD values between the models. The conventional model needs 3162 and 3486 times the number of simulated photons compared to the hybrid model in the weakly and strongly scattering cases, respectively. In conclusion, the hybrid particle-wave method is 3 orders of magnitude more efficient compared to the conventional detection model.

### 4.3. Simulation compared to experiment

Four different Intralipid dilution samples measured experimentally in a previously published work [5], described in more detail in Section 3.1, were simulated for a single focus position. Simulated as well as single-scatter theory depth profiles were fitted to experimentally measured depth profiles by fitting a proportionality constant A and a noise floor C as described in Section 3.2. All simulations were performed with the hybrid particle-wave detection model as it is 3 orders of magnitude more efficient compared to the conventional model, as reported in the previous section.

**Fig. 8.**
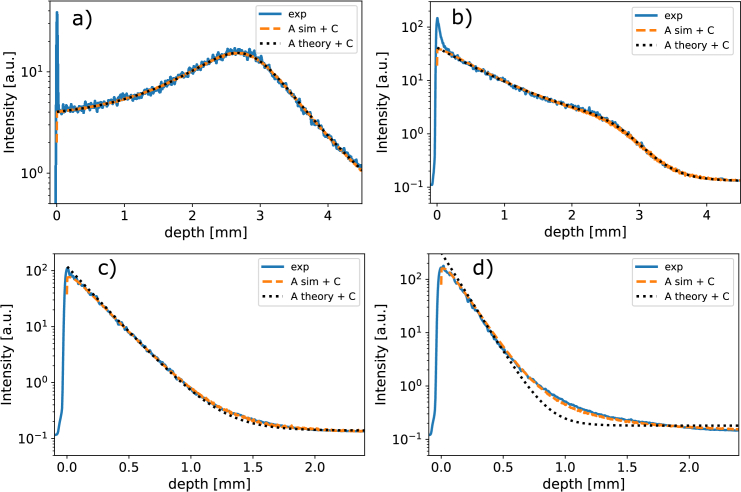
Depth profiles for Intralipid dilution samples I01, I1, I4, and I10, shown in **a**, **b**, **c**, and **d**, respectively. In each of the plots the experimentally measured depth profile is indicated by a solid blue line, the simulated depth profile is indicated by the orange dashed line and the single-scatter theory curve is indicated with a black dotted line. The simulated and single-scatter theory depth profiles were fitted to the experimentally measured depth profile with fixed optical properties and beam parameters and a free proportionality constant A and noise floor C , Eq. (22), see Section 3.2. All of the simulations were done with the hybrid particle-wave detection model, $2\times 10^{9}$ simulated photons, $w_{0}=15$ μ m, $z_{R}=750$ μ m, $\mu_{a}=0.15$ ${\mathrm{mm}}^{-1}$ and n=1.33 . The $\mu_{s}$ , g and $z_{f}$ depended on the sample with $\mu_{s}$ = 0.1, 1.0, 2.9 and 4.5 ${\mathrm{mm}}^{-1}$ , g = 0.33, 0.33, 0.3 and 0.24 and $z_{f}$ = 2.81, 2.71, 2.50 and 2.73 mm for I01, I1, I4 and I10, respectively.

The resulting depth profiles are shown in Fig. 8(a)-(d), corresponding to samples I01, I1, I4 and I10, respectively. The experimentally measured curves are indicated with solid blue lines, the simulated curves with orange dashed lines and the single-scatter theory fit curves with black dotted lines.

For all of the samples the simulated depth profiles and the experimentally measured profiles are in excellent agreement except for the strong specular reflection peaks at the air-sample interface for the I01 and I1 samples that are visible in the experiments and absent in the simulations. This is expected since specular reflection at the air-sample interface was not included in the simulations. For the more weakly scattering samples I01, I1 and I4 the single-scatter theory curves are also in good agreement with the experimentally measured curves. For the strongly scattering sample I10, however, the single-scatter theory curve does not fit the experimentally measured depth profile, which is particularly obvious around a depth of 1.0 mm.

We further notice that as the scattering coefficient increases the effect of the confocal function, observable as a peak in signal near the focus position, becomes less pronounced until it starts to disappear as the Lambert-Beer attenuation starts to dominate the signal. Additionally, due to the stronger Lambert-Beer attenuation the experimental noise floor is reached at shallower depths for higher scattering coefficients. This explains why in Fig. 8(c) the single-scatter theory fits simulated and experiment depth profiles rather well while in Fig. 6(b) the single-scatter theory starts to deviate from the simulated signal around 1 mm, despite Fig. 8(c) and Fig. 6(b) having similar scattering coefficients of 2.9 and 3.0 ${\mathrm{mm}}^{-1}$ , respectively. In short: in the experimental situation of Fig. 8(c) the noise floor is reached before the signal starts to deviate from single-scatter theory, while in the simulations of Fig. 6 there is no noise floor and we can observe deviations from single-scatter theory at depths larger than 1 mm.

## 5. Discussion and conclusion

In this work, we have developed and compared two distinct Monte Carlo OCT detection models, a conventional photon detection model and a hybrid particle-wave detection model based on spherical waves from a photon’s last scatter position. In order to characterize the noise on the simulations properly, we evaluated the normalized root mean square deviation (NRMSD) of the simulations with respect to a ground truth. By generating depth profiles free of statistical noise as ground truths using simulations with a large amount of photons and calculating the NRMSD between these ground truth depth profiles and simulated depth profiles as a function of the number of simulated photons, we showed that the hybrid particle-wave model is 3 orders of magnitude more efficient compared to the conventional model.

MC simulations were validated by simulating four experimentally measured Intralipid dilution samples with scattering coefficients $\mu_{s}$ ranging from $\mu_{s}=0.1$ ${\mathrm{mm}}^{-1}$ to $\mu_{s}=4.5$ ${\mathrm{mm}}^{-1}$ . The simulated depth profiles matched the experimentally measured depth profiles well across the whole range of sample scattering coefficients while the single-scatter theory matches the experimentally depth profiles well except for the strongly scattering sample case of I10. The scattering coefficients of the simulated depth profiles that matched the measured depth profiles the best for the Intralipid dilution samples I01 and I1 were equal to those of Aernouts [15,16] and followed the linear relation expected in the case of independent scattering. The scattering coefficients simulated for samples I4 and I10 were much lower than expected for a linear relationship between concentration and $\mu_{s}$ , therefore showing dependent scattering at higher concentrations. However, the scattering coefficients from the simulations for I4 and I10, 2.9 ${\mathrm{mm}}^{-1}$ and 4.5 ${\mathrm{mm}}^{-1}$ , respectively, were lower than those given by Aernouts in case of dependent scattering, 3.2 $m^{-1}$ and 5.5 ${\mathrm{mm}}^{-1}$ , respectively. The discrepancy between the simulated scattering coefficients that matched best to the experimental OCT measurements and those reported by Aernouts could be due to experimental uncertainties or to the phase function used in our simulations. Although the Henyey-Greenstein phase function as used in the simulations is able to generate the expected scatter anisotropy g , the actual phase function of Intralipid is different from the Henyey-Greenstein phase function [17]. It is also possible that the experimentally reported values by Aernouts are not accurate.

In our simulations, we assumed a refractive index step from air (n = 1) to water (n = 1.33), which leads to a critical angle of about $50^{\circ }$ . Due to the design of the code, photons hitting the surface interface were detected, and even in the case of total internal reflection, the simulation was terminated, and the total internally reflected photons were discarded. During the simulations, we evaluated Eq. (14). For very shallow angles ( $\theta \approx 90^{\circ }$ ), $P_{\text{adj}}$ explodes, leading to huge spikes in the simulated profiles. The critical angle prevented these spikes in the depth profiles by limiting $\theta <50^{\circ }$ .

The signal strength of the simulations in Fig. 6 represent the total detected energy, where a photon at launch had energy unity. We hypothesize that the better NRMSD performance of the hybrid model can be attributed to the larger number of simulated photons leaving the sample that contribute to the total energy, but each photon contributes a smaller energy value to the total detected energy, improving the statistics, i.e., reducing the variance. In the conventional model fewer simulated photons leaving the sample contribute to the total energy, but each photon contributes a larger energy value to the total detected energy. increasing the variance. The key contributor to this effect is the overlap integral of the spherical wave with the SMF mode. For the conventional model the photons leaving the sample at large angles are rejected by $W_{dir}$ in Eq. (9), while these photons still contribute to the detected energy in the hybrid model by interpreting these photons as a sampling of the spherical wave probability.

When comparing the total detected energy between the hybrid and conventional detection models the hybrid model has an intensity that is about a factor of 0.4 to 0.5 times lower for the same number of simulated photons, see Fig. 6. We attribute this difference to the cut-off angle of $50^{\circ }$ that we introduced to take into account total internal reflection at the medium-air interface, which reduces the surface of the half sphere sampling the spherical wave probability by approximately 0.4 . We hypothesize that the cut-off angle did not affect the conventional model, since these large angles are rejected by the angle acceptance probability $W_{dir}$ of the SMF (last exponential term in Eq. (9)).

The scaling behaviour of the SNR performance of MC simulations as $\sqrt{N}$ is often implicitly assumed. Figure 7 explicitly demonstrates this behaviour over more than two decades for both the conventional and hybrid models.

Although in this work only homogeneous media were simulated, both the conventional and hybrid particle-wave detection model can be used in more complex sample geometries including different media with different optical properties and shapes. In cases of more complex geometries with varying n and $\mu_{t}$ , Eq. (3) and Eq. (13) need to be used instead of their simplified results for homogeneous media, Eqs. (4),(14).

In conclusion, we have developed an efficient Monte Carlo OCT simulation method that could prove useful for the investigation of robust attenuation coefficient extraction methods for OCT in cases where single-scatter theory fails, such as for strongly scattering tissues or at large tissue depths. Furthermore, our fast Monte Carlo OCT simulations can be used to understand and develop methods to correct for the confounding effects of layers and other inclusions, such as blood vessels, on the determination of the OCT attenuation coefficient, as well as to investigate the effect of the phase function and anisotropy on OCT signals.

## Figures and Tables

**Fig. 9.**
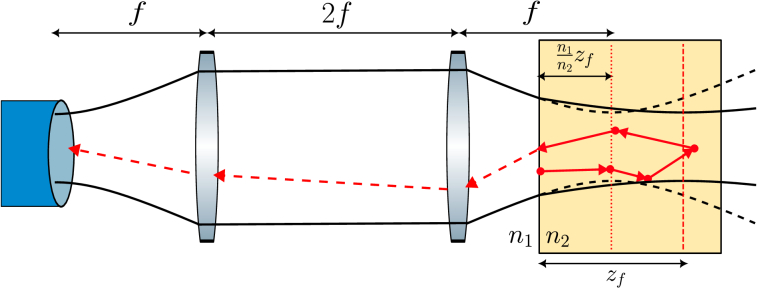
Sketch of (virtual) optical system with simulated sample used to propagate backscattered photons to SMF surface. The system consists of a SMF, blue cylinder, that functions as both the source of a Gaussian beam and detector of the photons backscattered from the sample, a scattering sample that is simulated in MCX, the sand coloured rectangle, and a 4f system which focuses the beam from the SMF into the sample and in the other direction focuses backscattered photons from the simulated sample on the SMF. The solid black lines indicate the (Gaussian) beam in different parts of the system, note the change in the Gaussian beam at the sample interface due to change in refractive index between sample and surrounding medium. The dashed black lines in the simulated sample indicate the shape of the Gaussian beam if there was no sample or if the sample had the same refractive index as the surrounding medium. The solid red arrows indicate propagation and scattering in the simulation by MCX ending at the sample interface, refraction at the interface and propagation to the SMF detector indicated by dashed red arrows and implemented via the ABCD ray transfer matrix method. The lenses have a focal length of f and the beam parameters in the sample are those used in the MCX simulation $w_{0}$ , $z_{f}$ and $z_{R}$ , which are the beam waist radius at the focus, distance from sample surface to the focus and the Rayleigh range respectively. The Gaussian beam parameters of the black dashed line are $w_{0}^{\prime }=w_{0}$ , $z_{f}^{\prime }=\frac{n_{1}}{n_{2}}z_{f}$ and $z_{R}^{\prime }=\frac{n_{1}}{n_{2}}z_{R}$ .

## Data Availability

Data underlying the results presented in this paper are not publicly available at this time but may be obtained from the authors upon reasonable request.

## A. Appendix

### A.1. Propagating photons to the SMF surface

For the conventional detection method discussed in Section 2.2.1 the MCX backscattered photons need to be propagated from the sample surface to the SMF surface and the position and direction of the photons at the SMF surface need to be determined to calculate their detected intensities, see Eqs. (7),(9). Here we discuss the method used to determine the position and direction at the SMF surface from the position and angle of the MCX simulated photon at the sample interface. At the SMF surface the Gaussian beam parameters are defined by $w_{0}^{\prime }$ , $z_{f}^{\prime }=0$ and $z_{R}^{\prime }$ which are the waist radius at focus, distance to the focus from the SMF surface and the Rayleigh range of the SMF beam, respectively. The beam parameters in the sample in the MCX simulation are then given by $w_{0}=w_{0}^{\prime }$ , $z_{f}$ and $z_{R}=\frac{n_{2}}{n_{1}}z_{R}^{\prime }$ which are the waist radius at focus, distance to the focus from the sample surface and the Rayleigh range of the beam in the sample with $n_{1}$ , $n_{2}$ being the refractive index of the surrounding medium and the simulated sample, respectively. The position and angle of the MCX simulated photon at the sample surface will be indicated by x and θ respectively while the same parameters defined at the SMF surface will be indicated with $x^{\prime }$ and $\theta^{\prime }$ . To propagate the backscattered photons from the sample interface to the SMF surface we used the ABCD ray transfer matrix method based on a 4f configuration with at one end the SMF, and at the other end the sample surface and a change in the refractive index between the sample ( $n_{2}$ ) and surrounding medium ( $n_{1})$ as shown in Fig. 9.

The ABCD ray matrix for the propagation of backscattered photons is then given by

(26)
$$\left[\begin{array}{cc} 1 & f\\ 0 & 1 \end{array}\right]\left[\begin{array}{cc} 1 & 0\\ -\frac{1}{f} & 1 \end{array}\right]\left[\begin{array}{cc} 1 & 2f\\ 0 & 1 \end{array}\right]\left[\begin{array}{cc} 1 & 0\\ -\frac{1}{f} & 1 \end{array}\right]\left[\begin{array}{cc} 1 & f-\frac{n_{1}}{n_{2}}z_{f}\\ 0 & 1 \end{array}\right]\left[\begin{array}{cc} 1 & 0\\ 0 & \frac{n_{2}}{n_{1}} \end{array}\right]=\left[\begin{array}{cc} -1 & z_{f}\\ 0 & -\frac{n_{2}}{n_{1}}, \end{array}\right]$$

with $z_{f}$ being the distance from the sample surface to the focus of the beam in the sample. Note when the $x^{\prime }$ and $\theta^{\prime }$ resulting from this matrix and $w_{0}$ and $z_{R}^{\prime }$ are plugged in Eq. (9) the refractive index fraction in front of $\theta^{\prime }$ and $z_{R}^{\prime }$ are each others inverse and cancel out resulting in an expression that only depends on values from the MCX simulations; x , θ , $w_{0}$ , $z_{R}$ and $z_{f}$ .

### A.2. Integrals power transmission coefficient

Here we provide a detailed calculation of the power transmission coefficient T , Eq. (10). The center of the coordinate system (0,0,0) is the position where the optical axis transects the sample surface (See Fig. 5). We calculate the overlap integral on the sample surface $\left(x_{p},y_{p},0\right)$ perpendicular to the SMF optical axis. The focus is at a distance $z_{f}$ from the sample surface inside the medium. The power transfer coefficient is then expressed by

(27)
$$T=\frac{{\left|\iint E_{SMF}^{*}(x_{p},y_{p},0)E(x^{\prime },y^{\prime },z)dx_{p}dy_{p}\right|}^{2}}{\iint {\left|E_{SMF}(x_{p},y_{p},0)\right|}^{2}dx_{p}dy_{p}\iint {\left|E(x^{\prime },y^{\prime },z)\right|}^{2}dx_{p}dy_{p}},$$

where $x_{p},y_{p}$ are the lateral coordinates with respect to the optical axis in the overlap plane and defining $x^{\prime }=x-x_{p}$ , and $y^{\prime }=y-y_{p}$ , where (x,y,z) is the location of the spherical wave source. Finally, $E_{SMF}(x_{p},y_{p},0)$ and $E(x^{\prime },y^{\prime },z)$ are the SMF field mode and E-field of the spherical wave respectively and are given below. The complex conjugate of the SMF mode is approximated by a Gaussian beam and is given by

(28)
$$E_{SMF}^{*}(x_{p},y_{p},0)=\frac{w_{0}}{w(z_{f})}e^{-\frac{x_{p}^{2}+y_{p}^{2}}{w{\left(z_{f}\right)}^{2}}}e^{i\left(kz_{f}+k\frac{x_{p}^{2}+y_{p}^{2}}{2R(z_{f})}-\psi (z_{f})\right)},$$

with

(29)
$$w(z_{f})=w_{0}\sqrt{1+{\left(\frac{z_{f}}{z_{R}}\right)}^{2}},$$

(30)
$$\frac{1}{R(z_{f})}=\frac{z_{f}}{z_{f}^{2}+z_{R}^{2}},$$

and

(31)
$$\psi (z_{f})=\arctan\left(\frac{z_{f}}{z_{R}}\right).$$

The E-field of the spherical wave is given by

(32)
$$\begin{array}{rl} E(x^{\prime },y^{\prime },z) & =\frac{e^{-ikr^{\prime }}}{\left|r^{\prime }\right|},\\ & \approx \frac{e^{-ikz}}{\left|z\right|}e^{-\frac{ik}{2z}\left(x^{\prime 2}+y^{\prime 2}\right)}, \end{array}$$

where $r^{\prime }=r-r_{p}=(x-x_{p},y-y_{p},z)$ and the second line is the small angle or paraxial approximation of the spherical wave.

Calculating the normalisation integrals in the denominator of T , starting with the Gaussian beam we get

(33)
$$\int_{-\infty }^{\infty }\int_{-\infty }^{\infty }{\left|E_{SMF}(x_{p},y_{p},0)\right|}^{2}dx_{p}dy_{p}=\frac{\pi w_{0}^{2}}{2}.$$

For the integral of the normalisation factor for the spherical wave the unapproximated spherical wave needs to be used since the paraxial approximation results in a diverging integral. This results in

(34)
$$\int_{-\infty }^{\infty }\int_{-\infty }^{\infty }{\left|E(x^{\prime },y^{\prime },z)\right|}^{2}dx_{p}dy_{p}=2\pi .$$

The absolute value squared of the overlap integral, this time using the small angle approximation of the spherical wave, gives the following result

(35)
$${\left|\iint E_{SMF}^{*}(x_{p},y_{p},0)E(x^{\prime },y^{\prime },z)dx_{p}dy_{p}\right|}^{2}=\lambda^{2}\frac{w_{0}^{2}}{w{\left(z^{\prime }\right)}^{2}}e^{-2\frac{x^{2}+y^{2}}{w{\left(z^{\prime }\right)}^{2}}},$$

where $z^{\prime }=z-z_{f}$ . Finally, combining the terms in the numerator and denominator of T we get the result

(36)
$$T=\frac{\lambda^{2}}{\pi^{2}w_{0}^{2}}\frac{w_{0}^{2}}{w{\left(z^{\prime }\right)}^{2}}e^{-2\frac{x^{2}+y^{2}}{w{\left(z^{\prime }\right)}^{2}}}={\left(\frac{w_{0}}{z_{R}}\right)}^{2}\frac{w_{0}^{2}}{w{\left(z^{\prime }\right)}^{2}}e^{-2\frac{x^{2}+y^{2}}{w{\left(z^{\prime }\right)}^{2}}},$$

which is equal to the result as used in Eq. (11).
